# The evolving Japanese: the dual structure hypothesis at 30

**DOI:** 10.1017/ehs.2020.6

**Published:** 2020-02-24

**Authors:** Mark J. Hudson, Shigeki Nakagome, John B. Whitman

**Affiliations:** 1 Eurasia3angle Research Group, Max Planck Institute for the Science of Human History, Kahlaische straße 10, 07745 Jena, Germany; 2 School of Medicine, Trinity College Dublin, 150-162 Pearse Street, Dublin, Ireland; 3 Department of Linguistics, Cornell University, 203 Morrill Hall, Ithaca, NY 14853, USA

**Keywords:** Agricultural dispersals, Bronze Age migrations, Japanese, Ainu, national identity

## Abstract

The population history of Japan has been one of the most intensively studied anthropological questions anywhere in the world, with a huge literature dating back to the nineteenth century and before. A growing consensus over the 1980s that the modern Japanese comprise an admixture of a Neolithic population with Bronze Age migrants from the Korean peninsula was crystallised in Kazurō Hanihara's influential ‘dual structure hypothesis’ published in 1991. Here, we use recent research in biological anthropology, historical linguistics and archaeology to evaluate this hypothesis after three decades. Although the major assumptions of Hanihara's model have been supported by recent work, we discuss areas where new findings have led to a re-evaluation of aspects of the hypothesis and emphasise the need for further research in key areas including ancient DNA and archaeology.

## Graphical abstract

**Figure:**
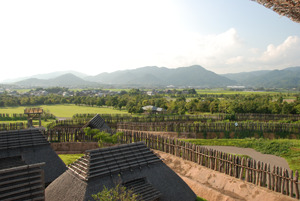

**Media summary:** The ‘dual structure hypothesis’ of two genetic layers in the population history of the Japanese archipelago still remains widely accepted after three decades, but new research is starting to suggest more complex social relations between Neolithic (Jōmon) and Bronze Age (Yayoi) peoples.

## Introduction

The population history of Japan has been one of the most intensively studied anthropological problems anywhere in the world. European interest in Japanese origins dates back to the first contacts with the country in the sixteenth century (Kreiner 1993). The introduction of anthropology and archaeology in the late nineteenth century provided new ways of studying the peoples of the archipelago and growing attention was paid to the so-called ‘Ainu problem’ (Sternberg 1929), which stemmed from the then widespread view that the Ainu were a ‘lost race’ of Caucasoids who had somehow ended up as an isolated island (*Rasseninsel*) within a Mongoloid sea (Koganei 1894; von Baelz 1911). For many Western visitors of the late nineteenth and early twentieth centuries, Japan's rapid success in modernisation seemed at odds with its non-European racial identity but, through the Ainu, writers such as W.E. Griffis (1843–1928) ‘claimed the Japanese for the Caucasians, creating in them a white tribe of Asia’ (Low 1999, p. 210).

Western insistence on the ‘mixed’ roots of the Japanese came as a shock to native scholars, who quickly developed theories emphasising the uniqueness of the Japanese race (Oguma 2002). By the 1970s, although a few Western anthropologists such as Howells (1966) and Turner (1976) continued to argue for ‘replacement’ theories, the ‘transformation’ approach – whereby the Neolithic Jōmon people evolved into the modern ‘mainland’ Japanese without significant immigration – was widely accepted (Hasebe 1940; Suzuki 1969, 1981; cf. Nanta 2008). Over the 1980s, however, several new developments in biological anthropology made it increasingly difficult to ignore an important role for immigration in the Bronze Age Yayoi period (900 BC to AD 250). The growing use of both cranial and dental nonmetric traits in biodistance analyses was significant because such traits are thought to be under less environmental selection than metric ones (Dodo and Ishida 1987). Somatometric and classical genetic markers had long suggested an east–west cline in the Japanese archipelago consistent with immigration from the west, and this was now further supported by new genetic work, including studies on dogs and mice (Horai *et al*. 1989; Hanihara 1991). Another influential analysis supporting immigration was Hanihara's (1987) demographic modelling which estimated a contribution of between 400,000 to over 1 million immigrants into Japan from the beginning of the Yayoi to the eighth century AD. By the early 1980s, Kazurō Hanihara (1927–2004), the leading Japanese physical anthropologist of his generation, was already writing about a ‘new’ theory of Japanese origins. Hanihara (1984a) emphasised the difference between two physical types in Japan, which he termed ‘Palaeo-Mongoloid’ and ‘Neo-Mongoloid’, admitting that this classification had similarities to earlier work by von Baelz (1883, 1885, 1911). Here, Hanihara managed to combine classical morpho-typological approaches with new techniques, a merger which without doubt led to the wide acceptance of his hypothesis. According to Nanta (2008, p. 42), Hanihara (1984b) had already used the Japanese term *nijū-kōzō moderu*, which was later translated as ‘dual structure model’. While the essential conclusions of the dual structure hypothesis were found in writings such as Hanihara (1985), it was not until 1991 that the theory was presented in an integrated way under the English label ‘dual structure’.

**Figure 1.**
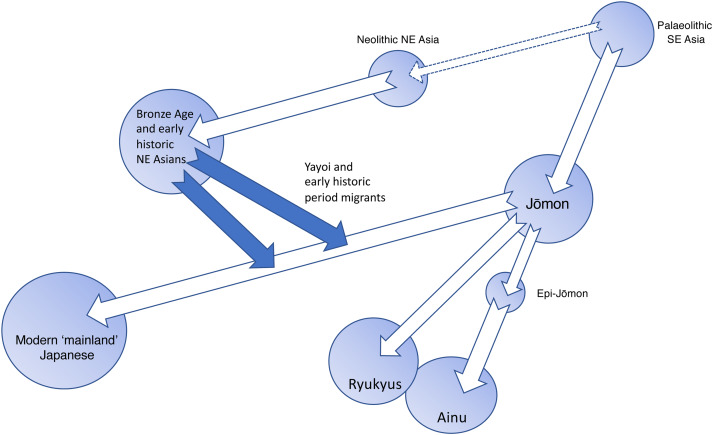
The dual structure hypothesis of the population history of the Japanese Islands. Based on Hanihara (1991) with amendments described in the text.

Figure 1 summarises Hanihara's dual structure hypothesis with two major amendments to the original. First, while Hanihara described Yayoi-period migrants as deriving from ‘modern’ Northeast Asians, the meaning of ‘modern’ in this context is unclear. The source populations for post-Jōmon immigration to Japan are better described as ‘Bronze Age and early historic NE Asians’. Secondly, while Hanihara emphasised migration to Japan in the Yayoi period, it is clear from historical records that substantial population movements from the Korean peninsula continued during the following Kofun and Nara periods (250–794 AD) and we have thus described these as ‘Yayoi and early historic period migrants’. While this figure *per se* refers only to the human biological history of the archipelago and not to linguistic or cultural phenomena, the dual structure hypothesis has been enormously influential in Japanese studies as a whole. In this paper we discuss the reception of this theory in Japan before looking at how new research has confirmed or changed Hanihara's original model.

## The dual structure hypothesis and national discourse in modern Japan

Hanihara (1991) is the most highly cited scientific paper on the population history of Japan by a Japanese scholar. According to Google Scholar (accessed 7 January 2020), Hanihara (1991) has been cited 399 times and a 1994 Japanese translation of the same paper on a further eight occasions. Since Google Scholar does not include many books and journals published in Japanese, the actual total is likely to be higher. The article has been cited in research in a wide range of disciplines, including (in order of citation) biological anthropology, genetics, Japanese studies and medical research. It is important to note the influential role of the paper in broader debates over Japanese origins. The dual structure hypothesis included several significant changes from previous research. Controversy over whether the Ainu were the original Stone Age inhabitants of the archipelago had begun with the first archaeological excavation in Japan in 1877 (Darwin and Morse 1880; Dickins 1880a, 1880b; Nishioka and Schenck 1937). Early studies regarded the Ainu as a Caucasoid isolate, unconnected to the Japanese and, even as late as the 1960s, it was believed that ‘the participation of the Ainu was relatively insignificant in the ethnic formation of the Japanese people’ (Ishida 1963, p. 6). One of Hanihara's major contributions was to argue that ‘the population history of the Ainu had an intimate relationship to that of the non Ainu Japanese’ (Hanihara 2000). Hanihara also expanded the problem of the ‘origins of the Japanese’ to a much wider geographic scale and later research used this insight to investigate how the dual structure hypothesis relates to human dispersals in Asia and the Americas (e.g. Brace *et al*. 2001; Matsumura and Hudson 2005; Adachi *et al*. 2011; Matsumura *et al*. 2019).

With its emphasis on immigration, the dual structure hypothesis can be seen as a critique of nationalist theories of Japanese racial homogeneity. At the same time, however, Hanihara followed a long tradition within Japanese anthropology of emphasising racial and, by implication, cultural amalgamation (*kongō*) and assimilation (*dōka*), terms which signify the process by which the diverse roots of the Japanese came, over time, to form one ‘unique’ (*koyū*) whole (Pai 1999). Although Hanihara used the translation ‘dual structure’, the original Japanese *nijū* draws on the concept of stratigraphic *layering*, reflecting a central problem in Japanese thought since the early twentieth century of how to understand the coexistence of old cultural forms within a rapid lurch towards modernity. Perhaps the most influential response to this problem was the concept of *jūsōsei*, or ‘stratigraphic layering’, developed in the 1930s by philosopher Tetsurō Watsuji (1889–1960). In this scheme, new layers were placed on top of the old but did not replace them. In an attempt to ‘overcome’ the contradictions of modernisation, Watsuji emphasised how ‘*jūsōsei* made possible a structure that housed all of the layers [of Japan's past] in the same space even though they signified different temporalities’ (Harootunian 2000, p. 254). This question of ethnic/cultural ‘layering’ has been central to anthropological discourse in Japan and folklorist Kunio Yanagita (1875–1962) was one of the first to recognise the importance of older ethno-cultural layers. Yanagita saw that ‘cultural unevenness, what he called *ainoko bunmei* (mixed civilisation), was not a temporary stage in an evolutionary narrative but a permanently entrenched condition that could be found throughout East Asia’ (Harootunian 2000, p. 31).

Against this background, archaeologists debated whether the Stone Age came to an end more or less simultaneously across Japan, or whether pockets of Stone Age Jōmon culture remained in eastern Japan as late as the Middle Ages (Darwin and Morse 1880; Dickins 1880a, b; Barnes 1990). The underlying question here was, to what extent were ‘primitive’ elements retained as Japanese culture evolved into ever higher forms? However, Hanihara's dual structure theory circumvented this whole debate by arguing for an apparently seamless integration of native and newcomer – although he did accept that the degree of admixture was lower in the extreme north and south of archipelago (see Nanta 2008). Philosopher Takeshi Umehara (1990) used Hanihara's work to define Japanese culture as an integrated ‘oval culture comprised of the harmonious opposition of two focal points, the forest culture that is Jomon and the paddy field culture that is Yayoi’, an influential conceptualisation which built on Nietzsche's opposition of Apollonian and Dionysian elements in Greek culture. While earlier researchers had usually seen the Japanese nation as beginning with the Yayoi (e.g. Ishida 1974), by the 1990s the Jōmon had been widely – yet uncritically – incorporated into a much longer Japanese tradition (Ikawa-Smith 1990, 1995; Hudson 2003; Kobayashi 2018), even though that argument contradicted the dual structure hypothesis in many respects. As discussed below, these assumptions about the seamless layering of admixture in prehistoric Japan have hindered Japanese scholarship from understanding the social processes behind the dual structure hypothesis.

## Evaluating the dual structure hypothesis

### Biological anthropology

The first critique of the dual structure theory came from Okinawa. Hanihara had argued that Ryukyuans retain significant Jōmon genetic heritage with little Yayoi/Japanese admixture. However, Hudson (1994) noted that since the Ryukyuan languages of Okinawa are closely related to Japanese, there must have been language replacement in the Ryukyu Islands in the post-Jōmon period. Given that demic expansion seems to have been the primary cause behind the spread of Japonic in mainland Japan, Hudson (1994, 1999) proposed that a similar process had been at work in the Ryukyus. Support for this suggestion came from anthropologists who found that Ryukyuan populations cluster more closely with mainland Japanese and are rather distant from the Ainu in cranial analyses (Dodo et al. 1998, 2000; Pietrusewsky 2010). The genomic analysis by Jinam *et al*. (2012) supports both perspectives under an assumption that the Ainu are direct descendants of the Jōmon. Jōmon ancestry may be less prevalent in Ryukyuans as compared with the Ainu but it is still large enough to characterise Ryukyuans genetically compared with mainland Japanese. Recent ancient genomic studies have directly confirmed this by showing that Ryukyuans have much higher Jōmon ancestry than mainland Japanese (Kanzawa-Kiriyama *et al*. 2017, 2019; McColl *et al*. 2018). Furthermore, even within the people living in the Ryukyu Islands, there is population stratification (i.e. differences in frequencies of genetic variants between the populations at a genome-wide scale) between Okinawa and the Miyako Islands, which is unlikely to be due to gene flow from Aboriginal Taiwanese to Miyako Islanders (Sato *et al*. 2014). Rather, this pattern was more likely shaped by isolation with a weak but continuous migration between the populations for the last 2000–3000 years. Therefore, the amount of Jōmon ancestry possessed by people living in the Ryukyu Islands still remains unclear, although this can be addressed in the near future with the increasing scale of whole-genome data.

Another important re-evaluation of the dual structure hypothesis came from proposals of significant intermarriage between Okhotsk and Ainu populations in Hokkaido (Sato *et al*. 2007). This is partially supported by Jeong *et al*. (2016), who found that the phylogenetic position of the Hokkaido Ainu forms an outgroup with respect to all East Asian populations. Furthermore, the Ainu exhibit a closer genetic affinity to people of northeast Siberia, such as the Itelmen and Chukchi, than to people of central Siberia, who have genetic affinity to all other East Asians. This suggests that the Ainu share ancestry with people living in northeast Siberia, who in turn share ancestry with Native Americans. Another mechanism that can explain this observation is recent gene flow between the Hokkaido Ainu and northeast Siberians. Ancient DNA data on Okhotsk people may shed further light on cultural interactions between Okhotsk and Ainu, as well as on the origins of the Okhotsk people.

Until recently, no genetic evidence had emerged to support Hanihara's (1991) hypothesis that the Jōmon people originated in Southeast Asia. In 2018, McColl and colleagues generated whole-genome sequence data from a 2500-year-old skeleton belonging to a Final Jōmon context in central Japan. An early divergence of the Jōmon lineage, coupled with high genetic affinity of the Jōmon individual with ancient Southeast Asians, provides strong support for a Southeast Asian origin of the Jōmon people (McColl *et al*. 2018). These results are also consistent with those from Jeong *et al*. (2016), suggesting that ancient and modern genomics can further facilitate our understanding of the complex population history of the East Asian continent and the Japanese archipelago.

With respect to the Jōmon–Yayoi transition in the central islands of Japan, Hudson (1999) attempted to reconcile Hanihara's hypothesis of a substantial (in fact dominant) continental contribution to the Japanese population with the farming/language dispersal hypothesis (Bellwood and Renfrew 2002). It is notable that other researchers who have attempted to relate the Yayoi expansion to farming dispersals have been less willing to accept Hanihara's view of a major movement of population from the continent (e.g. Miyamoto 2009). Hanihara was well aware of this resistance, noting that ‘Most … anthropologists and archaeologists used to believe that the number of migrants was very small or almost negligible. However, if we adopt various current evidence showing a large impact of migrants upon the indigenous Japanese people, this idea is hardly acceptable’ (Hanihara 1991, p. 247). With recent advances in genetic analysis, we are now in a position to assess which of these views is correct. What can we expect to see in genetic data if the dual structure theory is true? One point would be a close genetic relationship between Ainu and Ryukyu Islanders; those two populations should share a common ancestor earlier than the ancestor shared with modern Japanese. Classic genetic markers, including mitochondrial DNA, blood groups, cell enzyme, serum protein systems and Y chromosomes, all support this expectation (Hammer and Horai 1995; Horai *et al*. 1996; Omoto and Saitou 1997; Tanaka *et al*. 2004; Hammer *et al*. 2006; Matsukusa *et al*. 2010; Koganebuchi *et al*. 2012). The admixture proportion in the Japanese population as a whole has been estimated to be 65% from the Yayoi people and 35% from the Jōmon people (Horai *et al*. 1996). However, this interpretation is subject to the caveat that these classical data were derived from only a small number of genetic markers. In contrast, genomic data include hundreds of thousands of independent genetic loci, each of which has its own unique history shaped mainly by random genetic drift and demography. Therefore, such large genetic datasets enable reconstruction of population histories at finer scales, shedding light on evolutionary processes that have shaped patterns of genetic variation under complex demographic histories.

Population stratification between Ryukyu Islanders and other Japanese (i.e. differences in allele frequency between the local populations) was first confirmed by genome-wide data with approximately 140,000 single nucleotide polymorphisms that were collected from 7003 individuals (Yamaguchi-Kabata *et al*. 2008). A following study by Jinam *et al*. (2012) genotyped Hokkaido Ainu and Ryukyu Islanders, as well as mainland Japanese, with 1 million single nucleotide polymorphisms, and demonstrated different population clusters where the Ryukyu cluster is located in between the Ainu and mainland Japanese. Therefore, genome-wide data also support the dual structure hypothesis. However, the same population clusters of Ryukyu Islanders and the other Japanese shown in Yamaguchi-Kabata *et al*. (2008) could be explained by an alternative model in which all modern Japanese evolved from a single Jōmon ancestor and the following long-term isolation of the Ryukyuans could have developed their own genetic characters owing to strong genetic drift.

Although Hanihara (1991) has been the most influential theory on the origins of the modern Japanese over the past three decades, historically three main hypotheses have been proposed to explain this question: (a) transformation (Hasebe 1940; Suzuki 1969, 1981); (b) replacement (Howells 1966; Turner 1976); and (c) hybridisation (Kiyono 1949). The dual structure theory can be placed in the third category. Nakagome *et al*. (2015) applied an approximate Bayesian computation approach for genome-wide scaled datasets, and, for the first time, quantitatively evaluated the fit of these three models to the genomic data used in Jinam *et al*. (2012). Their results provided support for the hybridisation model, although, interestingly, better statistical support was given for a complex scenario that includes population stratification within the Jōmon rather than a simple model of a single Jōmon lineage. This raises new questions of the extent to which Jōmon people were geographically differentiated and whether the impacts of Yayoi migration were uniform across the Japanese archipelago. Earlier studies of cranial morphology had suggested regional and chronological diversity within Jōmon populations (Kondo *et al*., 2017), but differing regional impacts of Yayoi-period migrants remain poorly understood.

### Historical linguistics

A major question raised by the dual structure hypothesis from a linguistic standpoint is the continental affiliation, if any, of the Japonic language family. Notwithstanding some dissenting voices such as Vovin (2010), evidence for a genealogical relationship between Japanese and Korean is gaining acceptance among linguists (Whitman 1985, 2012; Robbeets 2005; Unger 2009; Francis-Ratte 2016). It has long been understood that the relatively shallow time-depth of the Japanese–Ryukyuan or Japonic family is not consistent with a scenario where Japonic diversification began at the outset of the Jōmon period (c. 14,500 BC). The traditional comparative method, applied to Old Japanese and Ryukyuan, indicates a date for the divergence of proto-Ryukyuan somewhat prior to the eighth century. Hattori's (1953) glottochronological study estimated a date of AD 500 for the divergence of the ancestors of Early Middle Japanese and Shuri Ryukyuan. More recently, Lee and Hasegawa (2011) used a Bayesian phylogenetic analysis to arrive at a median date of 2182 BP for the initial divergence of the Japonic languages. All of these dates vastly postdate the beginning of the Jōmon and are significantly later than recent archaeological dates for the inception of the Yayoi period. In fact, given new dates for the beginning of Yayoi (see below), none of these dates correspond to an event (or cluster of events) in the archaeological record. However, this is not surprising: linguistic techniques track when populations become sufficiently separated to innovate independently. The range between Hattori and Lee and Hasegawa's dates seems likely to reflect the probable gradual expansion of the proto-Japonic speaking population out of north Kyushu, an interpretation which may be supported by recent archaeological research discussed in the next section.

Linguists have developed hypotheses that parallel the transformation, hybridisation, and replacement scenarios described above. A linguistic counterpart of the transformation hypothesis was proposed by Kamei (1973), who claimed that Japonic originated as a Jōmon language in Kyushu and spread with the Yayoi expansion. Kamei's hypothesis was consistent with the then current view that the Yayoi involved little or no demic diffusion from the continent, but it has difficulty explaining the toponymic evidence for a ‘para-Japonic’ language or languages spoken on the Korean peninsula. A counterpart of the hybridisation model is Maher's (1996) ‘North Kyushu Creole’ hypothesis. According to this view, proto-Japonic is the result of contact between a continental population and indigenous Jōmon groups. The weakness of Maher's hypothesis is that there are few, if any, examples where the classical model of pidgin formation followed by creolisation applies to linguistic interactions between agricultural and hunter–gatherer populations. More typical of such interactions is the formation of ‘mixed’ languages such as Michif or Chinook Jargon (Thomason and Kaufman 1988), but such languages are often drastically simplified (as in the case of Chinook Jargon) –far beyond anything evident in the proto-Japonic lexicon or grammar – and do not outlive protracted contact between the two populations.

The whole field of archaeolinguistics has been missing from Japanese scholarship and naive comments about language prehistory in the archipelago are common in the literature. Environmental archaeologist Yoshinori Yasuda (2010, p. 169), for instance, claims that, ‘The area of distribution of Jōmon pottery means that a shared culture and language existed there’. Building on the ideas of the eighteenth-century nativist scholar Motoori Norinaga, Jōmon specialist Tatsuo Kobayashi (2018, p. 132) also argues that the Japanese language was already present in Jōmon times, insisting that ‘Japanese is not something that came from some other, distant region. Japanese was born on the stage of the Japanese archipelago. The Jōmon Yamato *kotoba* was already in existence in the Jōmon period and has been nurtured [here] for more than ten thousand years.’ Hudson (1994, 1999, 2002) was the first to apply archaeolinguistic theory to Japan, proposing that proto-Japonic arrived in the archipelago together with a Bronze Age migration from the Korean peninsula resulting in the development of Yayoi culture, and then spread with the Yayoi expansion (the latter point is in agreement with Kamei). The current absence of any non-Japonic language on the archipelago other than Ainu is due to language shift, that is, the replacement of Jōmon languages by Japonic. The important point here is that, while the genetic hybridisation model represented by the dual structure hypothesis is not incompatible with linguistic replacement, linguistic hybridisation is much rarer than genetic hybridisation. Creoles and mixed languages do arise, but only in specialised socioeconomic circumstances; in cases of demic diffusion, language shift is the norm.

Research in historical linguistics has continued to explore the farming/language dispersal hypothesis for the East Asian region (Robbeets and Savelyev 2017; Whitman and Hudson 2017). As our archaeological understanding of agricultural expansions in the region has become more detailed (Stevens and Fuller 2017; Leipe *et al*. 2019), it has become possible to develop more detailed correlations between language and farming dispersals (Whitman 2011; Miyamoto 2016; Robbeets 2017a, b; Li *et al*. in press; Nelson *et al*. in press; Robbeets *et al*. in press). However, while the broad outlines of the farming/language dispersal hypothesis applied to the Japanese Islands remain well understood, the case of Japan is complex, in part because the transition to full-scale farming occurs much later – already well into the Bronze Age – than the rest of Northeast Asia (Hudson 2019, in press).

Estimates of the date of the dispersal of proto-Ainu also give a date which is not consistent with the much older arrival of Jōmon culture in the archipelago. Hattori and Chiri's (1960) glottochronological study gave a date of 1050 BP for the divergence of proto-Ainu, while Lee and Hasegawa's (2013) Bayesian study arrived at a date of 1300 BP. Vovin's (1993) reconstruction of proto-Ainu includes both cultural and basic vocabulary that are loans from Old Japanese (eighth century AD), such as *kamuy ‘god/bear’ < OJ *kamwi* ‘god’, *pasuy ‘chopsticks’ < pJR *pasuj, as well as *pone ‘bone’ < OJ *pone* and *sippo ‘salt’ < OJ *sipo*. At the same time, Vovin's (2009) study of Ainu place names indicates an earlier broad distribution of proto-Ainu, ranging at least as far west as the Noto peninsula. These facts tell us that Ainu language is not the ‘pure’ survival of a localised Jōmon language. It is, in part, the product of contact with Japonic and was spoken over a wide area, suggesting that it may have had the nature of a contact language, spoken on the frontier between Yayoi and Jōmon cultures. The shallowness of the protolanguage that linguists are able to reconstruct must reflect some relatively recent event leading to the loss of cognate varieties that would have, had their descendants survived, allowed reconstruction of an older ancestor. Candidates for this event that match the dates proposed for proto-Ainu are the advent of Satsumon culture around the eighth century and the slightly later movement of that culture to Hokkaido (Crawford and Whitman 2019). The late Satsumon/early Ainu period expansions across Hokkaido and into Sakhalin around the twelfth century may have also been associated with the spread of a central or standard Ainu language variety (cf. Hudson 2018).

### Archaeology

The dual structure hypothesis models the biggest period of discontinuity in Japanese history, the Jōmon–Yayoi transition. However, Hanihara (1991) did not discuss how the biological admixture proposed in his hypothesis might have been achieved in social terms. While this should have been a job for archaeology, Japanese archaeologists rarely discuss Hanihara's model, in part because the whole topic of ‘ethnic’ origins became taboo in the postwar era (Hudson 2006), but also because Japanese archaeologists are suspicious of macro-scale models, preferring to focus on the diversity of regional trajectories. The ‘transformation’ theory of Suzuki (1969, 1981) and others was used by archaeologists such as Akazawa (1986), who analysed the origins of Yayoi agriculture as an extension of the varied adaptive processes of Jōmon culture, arguing that rice farming enabled Jōmon populations in western Japan to achieve a stable food supply. This essentially teleological approach continues to be employed in the literature, recently for example by Temple (2019) and Watanabe et al. (2019).

Hudson's (1999) social archaeology of the dual structure theory used the farming/language dispersal hypothesis to link agricultural expansions with the spread of Japonic and immigrant Bronze Age populations. However, archaeological research over the past two decades has necessitated a number of important changes to previous understandings of the Jōmon–Yayoi transition. The first is provided by radiocarbon dates which shift the beginning of the Yayoi period back to around 900 BC (Shoda 2010; Fujio 2011; Miyamoto 2018). This longer time span means that the social processes behind the spread of Yayoi populations need to be re-evaluated. While in the 1990s it was assumed that the Yayoi spread very quickly at the expense of Jōmon culture (Hudson 1990, 1999), a much more gradual and contested expansion now seems more likely. The idea – inherent in Hanihara (1991) but made explicit by popular writers such as Umehara (1990) – that the Yayoi spread quickly to admix with the Jōmon and form a Japanese culture essentially identical to that known from the premodern historical record needs to be completely re-assessed. Contrary to earlier accounts, the shift from Jōmon to Yayoi was slow and involved complex historical processes whereby Jōmon populations developed new economic niches which enabled them to retain some degree of autonomy from Yayoi farmers (Hoover and Hudson 2016; Segawa 2017; Hudson 2019, in press). For instance, the *Hizen Fudoki*, an eighth-century gazetteer, noted that in the Gotō islands of Nagasaki, ‘The facial features of seafarers living on these islands resemble those of the *hayahito* [Hayato]. … They speak an entirely different language from the other local residents [of Hizen Province]’ (Aoki 1997, p. 265). This text suggests that more than 1600 years after the start of the Yayoi, there were still populations in northwest Kyushu who retained a Jōmon language and phenotype. Similar groups were probably found in southern Kyushu as well (Hudson 1999). Such peoples were not, however, isolated and passively awaiting the arrival of rice and civilisation. The *Hizen Fudoki* notes that, in addition to exploiting fish and other marine products, the Gotō islanders also raised cattle and horses. As recently argued by Segawa (2017) and Hudson (2019), the transition from Jōmon to Yayoi involved a radical re-structuring of economic practices in the Japanese Islands. While immigrant farming populations did expand across the archipelago as predicted by Hanihara, native Jōmon groups also developed new niches based on trade, specialised fishing and maritime hunting, and even pastoralism. Thus, while it may still be possible to follow Hanihara in modelling *biological* admixture in terms of ‘Jōmon’ vs ‘Yayoi’, *cultural* interaction did not follow the binary framework suggested by the dual structure hypothesis. In fact, rather than a dual structure, the archaeological evidence suggests a much more diverse, *multicultural* context for the admixture between post-Jōmon and Yayoi. Furthermore, we have no reason to assume that the immigrant ‘Yayoi’ populations focused solely on agriculture; for various reasons, including a desire to escape the growing power of the state (cf. Scott 2017), many such individuals or groups may well have participated in post-Jōmon non- or para-agricultural practices.

The diversity of Yayoi culture has been increasingly emphasised by archaeologists over the past decade or so, yet there remains a reluctance to re-define the Yayoi beyond a Childean framework (Hudson 2019). For instance, Fujio (2013) explains the Yayoi as a culture which adopted irrigated wet rice cultivation as its basis of production and which engaged in ‘Yayoi rituals’ to maintain that production. However, this leads him to conclude that less than half of the Japanese archipelago fits his own definition. Like Fujimoto (1988) before him, Fujio links rice with a ‘central culture’ zone and dismisses the north and the south – regions of great importance to the dual structure hypothesis – as ‘blurred’ or ‘fuzzy’ cultural zones surrounding the Yayoi. Discussing the Neolithic transition in Europe, Robb (2013) has called this the problem of ‘cowboys and Indians’ – the assumption that there were two separate (‘Mesolithic’ and ‘Neolithic’) peoples. Although historians of colonial North America have written extensively about the ‘Middle Ground’ (White 2010), the concept of ‘fuzziness’ proposed by Fujimoto and Fujio represents a reluctance to consider any sort of pluralistic middle ground whatsoever.

On one level, the dual structure hypothesis is a broad-brush model and probably few Japanese archaeologists would deny its central claim that there was ‘admixture’ between Jōmon and Yayoi populations. Yet those same archaeologists typically produce highly detailed analyses of changes in pottery and other material culture over the Jōmon–Yayoi transition – sometimes at the level of decoration on individual pottery sherds (Okita 1993) – without referring explicitly to models such as Hanihara (1991). Based on continuities in material culture, archaeologists often make claims that, for example, in west Kyushu ‘almost no interbreeding occurred with travelers [sic] from the Korean peninsula during the Yayoi period’ (Nakazono 2011, p. 57). As noted above, there were important Jōmon continuities in the Yayoi period in western Kyushu yet, as has recently become clear in Europe (Shennan 2018), this does *not* mean that we can rule out gene flow from immigrant farmers. A recent aDNA analysis of two Late Yayoi individuals from the Shimomotoyama site (Nagasaki) has shown significant gene flow from immigrant populations in this region (Shinoda *et al*. 2019). The ancient DNA ‘revolution’ is shining new light on how the lack of integration between archaeological research and models of population change means that the archaeology of the dual structure hypothesis remains poorly explored even after three decades.

## Conclusions

Published in 1991, Kazurō Hanihara's dual structure hypothesis successfully crystallised a range of new research in Japanese biological anthropology in the 1980s. Since then, the hypothesis has provided a foundation for a variety of disciplines to model the population history of the Japanese archipelago, although both archaeologists and linguists in Japan have made few attempts to engage directly with Hanihara's work. With its emphasis on immigration, the dual structure hypothesis was in certain respects a radical departure from previous work, yet it is this very aspect which has been most controversial – and largely ignored by Japanese archaeology. Within biological anthropology, research over the past three decades has generally supported the dual structure hypothesis, with the important qualifications discussed above. To what extent Hanihara's hypothesis will be affected by new analyses in ancient DNA remains to be seen. Outside Japan, research in historical linguistics has provided growing evidence of the long-range genetic relationships of Japonic and the expansion of that family has been successfully modelled by the farming/language dispersal hypothesis, two areas of research which also support the dual structure hypothesis.

## Data Availability

All data used for this article can be found in the published literature cited in the references.
